# High expression of PDLIM5 facilitates cell tumorigenesis and migration by maintaining AMPK activation in prostate cancer

**DOI:** 10.18632/oncotarget.20981

**Published:** 2017-09-18

**Authors:** Xi Liu, Lu Chen, Hai Huang, Jian-Min Lv, Ming Chen, Fa-Jun Qu, Xiu-Wu Pan, Lin Li, Lei Yin, Xin-Gang Cui, Yi Gao, Dan-Feng Xu

**Affiliations:** ^1^ Department of Urinary Surgery, Ruijin Hospital, Shanghai Jiaotong University School of Medicine, Shanghai 200025, China; ^2^ Department of Urinary Surgery, Third Affiliated Hospital, Second Military Medical University, Shanghai 201805, China; ^3^ Department of Urinary Surgery, Changzheng Hospital, Second Military Medical University, Shanghai 200003, China

**Keywords:** PDLIM5, epithelial-mesenchymal transition, prostate cancer, migration, AMPK

## Abstract

PDZ and LIM domain 5 (PDLIM5) is a cytoskeleton-associated protein and has been shown to bind to a variety of proteins through its specific domain, thereby acting to regulate cell migration and tumor progression. Here, we found that PDLIM5 was abnormally upregulated in prostate cancer (PCa) tissues as compared with that in normal prostate tissue. ONCOMINE microarray data mining showed that PDLIM5 was closely correlated with the prognosis of PCa in terms of Gleason score, tumor metastasis and biochemical recurrence. Lentivirus-mediated short hairpin RNA (shRNA) knockdown of PDLIM5 inhibited cell proliferation and colony formation, arrested hormone independent PCa cells DU145 and PC-3 in G2/M phase, and induced apoptosis. Meanwhile, silencing PDLIM5 inhibited migration and invasion of tumor cells by reversing the mesenchymal phenotype and a similar result was confirmed in a xenograft nude mouse model. Finally, we found PDLIM5 plays a crucial role in regulating malignant tumor cell proliferation, invasion and migration by binding to AMPK and affecting its activation and degradation, and may therefore prove to be a potential oncogenic gene in the development and progression of PCa.

## INTRODUCTION

Prostate cancer (PCa) remains the most commonly diagnosed cancer and the leading cause of cancer-related mortality in men in the western world [1]. It is estimated that there will have been 1.7 million new cases annually worldwide by 2030 [2]. Early screening efforts have made it possible to detect PCa in the localized stage in most patients, thus providing a relatively favorable prognosis and long-term survival by using standard therapeutics. However, many patients eventually progressed to castration resistant prostate cancer (CRPC) and relapsed, although the appropriate use of androgen deprivation therapy (ADT) can retard disease progression in patients with locally advanced or metastatic PCa. There are limited therapies for these patients and the prognosis is usually poor. Cancer metastasis is the cause of 90% cancer-related deaths, and PCa is one of the most prevalent human cancers easily metastasizing to the bone [3]. Multiple trials on novel chemotherapies have proved that the benefit that CRPC patients, especially metastatic CRPC (mCRPC) patients, could gain from these new chemotherapies is limited [4]. Therefore, it is necessary to identify effective bio-molecular targets and investigate potential pathways underlying PCa metastasis for the sake of developing better strategies for the treatment and prevention in CRPC.

PDZ and LIM domain 5 (PDLIM5) is a 63 kDa cytoplasmic protein composed of a PDZ domain in the N-terminus and three consecutive LIM domains in the C-terminus. PDLIM5 was first discovered in 1996 by Kuroda et al [5] by using the yeast two-hybrid technique with PKC as bait protein. PDLIM5 is anchored to the actin cytoskeleton through its PDZ domain and recruits the actin filament-associated protein [6]. It is thought to be involved in cytoskeletal organization, cell lineage specification, organ development and oncogenesis [7, 8]. The functions of PDLIM5 have been studied mainly in relation to its functions in modulation of synaptic strength [9]. Therefore, large numbers of studies have focused on the association between PDLIM5 and psychiatric diseases such as schizophrenia and depression. Besides, PDLIM5 was considered necessary in cell migration by forming lamellipodia, and phosphorylation inactivation of PDLIM5 was reported to weaken the cell metastasis capacity by inactivating RAC1 [10]. According to recent studies, PDLIM5 may participate in the progression of many types of cancer including thyroid cancer [11], lung cancer [12], breast cancer [13] and gastric cancer [14]. Koutros et al [15] reported that PDLIM5 mRNA was overexpressed in PCa tissues by using gene chip detection. Ma et al [16] reported that PDLIM5 may prove to be a diagnostic molecular target for serum and urine assays for adjuvant diagnosis and have a potential value in predicting the risk of advanced PCa progression. Cell migration and invasion are known to be closely related to dynamic changes of the cytoskeleton, especially the actin-containing microfilament skeleton. The arrangement of cytoskeleton is a key factor in determining cell morphology and motility [17, 18]. Knowing that PDLIM5 is a cytoskeleton related protein and may play an important role in the invasion and metastasis of PCa cells, an understanding about the molecular mechanisms of PDLIM5 in PCa initiation, development and metastasis would help improve the treatment of PCa patients, even those in the CRPC stage.

AMP-activated protein kinase (AMPK) is an energy receptor that is activated during hypoxia, ischemia, glucose loss, and stress [19]. Studies have demonstrated that activation of AMPK is closely associated with tumor growth and proliferation, cell cycle, apoptosis, neovascularization and metastasis [20, 21]. In this study, we found that PDLIM5 was over-expressed in PCa tissues as compared with normal prostate tissues both by database-mining and experimental investigation. In addition, PDLIM5 may exert a physiological function by reacting with AMPK. So we silenced PDLIM5 gene in PC-3 and DU145 cells by using lentivirus expressing short hairpin RNA to observe the phenotype changes of cancer cells and uncover the underlying regulatory mechanism in the tumorigenesis of PCa of PDLIM5.

## RESULTS

### PDLIM5 is overexpressed in PCa tissues and correlate with Gleason score, prostate-specific antigen (PSA) and metastasis with database analysis

**Figure 1:**
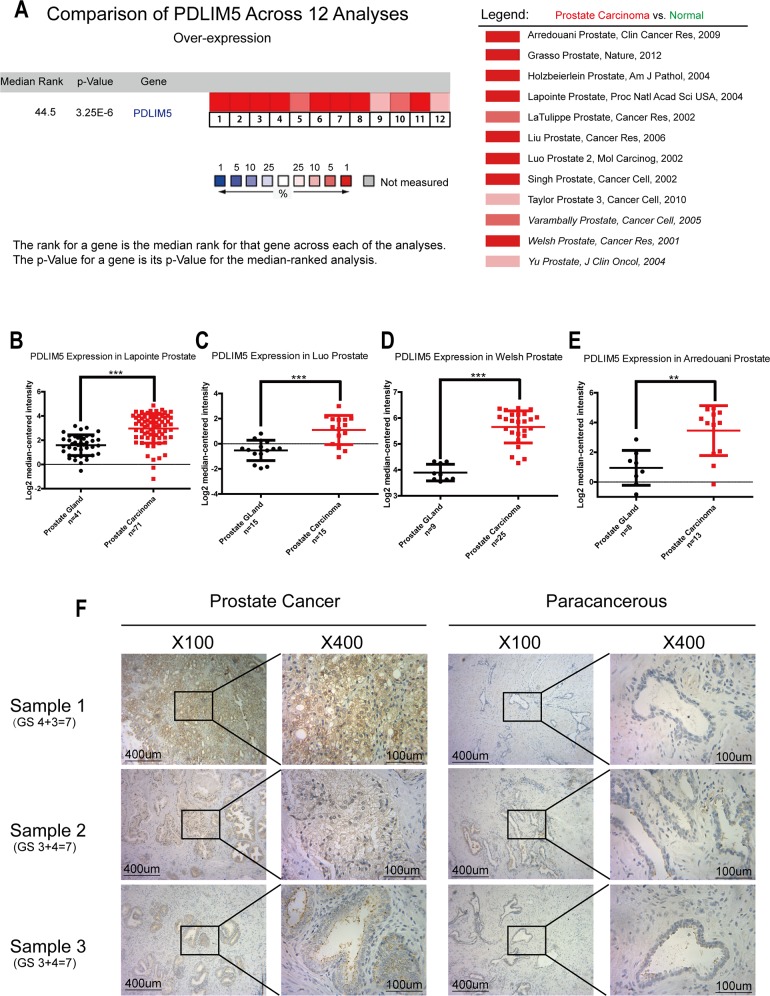
PDLIM5 expression in PCa *vs.* normal prostate gland was analyzed by using ONCOMINE microarray database and immunohistochemical method **(A)** Twelve microarray datasets regarding PDLIM5 mRNA expression in PCa vs. normal prostate gland were included in our meta-analysis. Data are shown as the median rank of PDLIM5 through each dataset analysis. P-value for PDLIM5 was presented using the median ranked analysis about PCa *vs.* normal tissues. **(B-E)** Datasets showed that PDLIM5 mRNA expression in PCa was significantly up-regulated as compared with normal tissues in Laponite, Luo, Welsh and Arredouani microarrays. Data are presented as Log2 median-centered intensity. **(F)** Representative immunohistochemical images are presented about PDLIM5 expression in PCa and paired paracarcinoma tissues. (^**^P< 0.01; ^***^P< 0.001).

**Figure 2:**
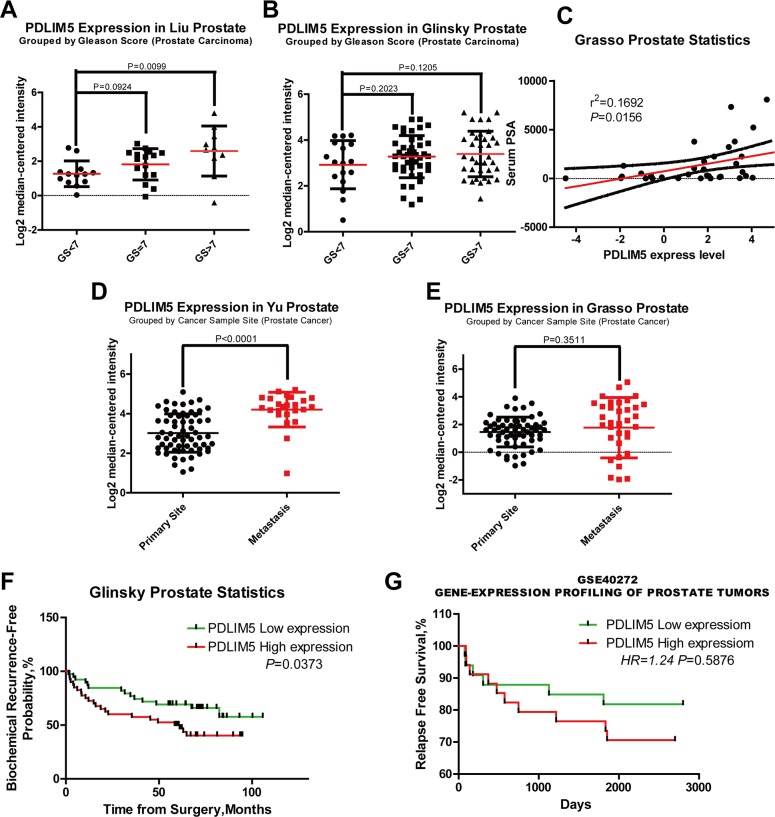
The expression of PDLIM5 was correlated with clinic pathologic features and clinical prognosis in ONCOMINE and PROGgene microarray database **(A, B)** High expression levels of PDLIM5 suggested a high Gleason score in Liu and Glinsky microarrays. **(C)** PDLIM5 mRNA expression was positively correlated with PSA mRNA expression in Grasso Dataset (r2 =0.1692 and P=0.0156). **(D, E)** Elevated expression of PDLIM5 mRNA in metastasis tissues was shown in Yu and Grasso microarray dataset; correlation between PDLIM5 expression and BCR-free probability **(F)** and relapse-free survival **(G)**. Analyses using Lapointe datasets (ONCOMINE), GSE40272 datasets (PROGGENE). Hazard ratio (HR) and P-value are shown in the images.

Twelve cases of database summary found that PDLIM5 mRNA level was elevated in cancer tissues versus prostate glands (Figure 1A), and representative database also showed that PDLIM5 expression level in PCa tissues was elevated as compared with normal tissues by using its value of log2 median-center intensity information (Figure 1B-1E). Immunohistochemical staining was used to analyze PDLIM5 expression in paired PCa specimens. The result verified high expression levels of PDLIM5 in PCa tissues and strong staining in high-grade PCa cells (Figure 1F). In PCa group, 38.6% of the specimens were strongly positive *vs.* 2.3% in paracarcinoma group (P<0.001) (Table 1). Furthermore, Liu Prostate of oncomine (Figure 2A) also revealed that PDLIM5 expression was significantly higher in PCa tissues with a greater Gleason score (Gleason grade<7 vs.=7: P=0.092;Gleason grade<7 vs. >7: P=0.01), and the Glinsky prostate (Figure 2B) showed the similar result. With respect to Grasso Prostate Statistics (Figure 2C), the expression level of PDLIM5 was positively correlated with serum PSA (spearman's correlation=0.17, P=0.02), further suggesting that the expression of PDLIM5 was related to the degree of malignancy of PCa. In metastasis, we also found that the expression of PDLIM5 in metastatic lesions was higher than that in primary site in Yu Prostate (Figure 2D, p<0.001) and Grasso Prostate (Figure 2E).

**Table 1:** IHC staining of PDLIM5 expression in prostate cancer tissues

	-~+	++	+++	Case	The rate ofstrong positive	*P^a^*	*P^b^*
**Carcinoma**	**1 (2.3)**	**26 (59.1)**	**17 (38.6)**	**44**	**38.6%**	**<0.001**	**<0.001**
**Paracarcinoma**	**10 (22.7)**	**33 (75.0)**	**1 (2.3)**	**44**	**2.3%**		

^a^ Mann-Whitney U test; ^b^ Wilcoxon signed-rank test.

### PDLIM5 high expression predicts poor prognosis in PCa patients

Metastasis and biochemical relapse are important factors affecting survival of PCa patients. We used oncomine and PROGgene datasets to explore the clinical data and found in Glinsky (oncomine) dataset (Table 2) that PDLIM5 overexpression was positively correlated with capsular invasion (P=0.009), extracapsular extension (P=0.056) and biochemical recurrence (BCR) (P=0.054). Kaplan-Meier survival analysis showed that elevated expression of PDLIM5 indicated a lower proportion of BCR-free survival or relapse-free survival (RFS) in both Glinsky and GSE40272(PROGgene) datasets (P=0.0373 and P=0.5876, respectively) (Figure 2F and 2G). Finally, high expression of PDLIM5 reducing OS was conclude by TCGA database (PROGgene, Supplementary Figure 3, Hazard ratio (HR) = 1.6, P=0.226). We also used univariate and multivariate Cox regression to analyze BCR by Glinsky dataset (Table 3). Univariate analysis indicated that high PDLIM5 expression (HR =2.002, P=0.041), high PSA level (HR=2.107, P=0.026), Gleason score>7 (HR=3.388, P<0.001), high grade of N stage (HR=3.704, P=0.033) and Seminal Vesicle Invasion (HR = 3.825, P=0.001) were risk factors for BCR of PCa patients. By adjusting multivariate analysis, Gleason Score (Gleason Score (HR = 2.883, P=0.010) and high PDLIM5 expression (HR = 2.792, P=0.007) were considered as the independent hazard factors affecting BCR in PCa patients.

**Table 2:** PDLIM5 expression and patient characteristics of glinsky prostate dataset

	No. Pts	No. PDLIM5		*p* Value
		Low	High	
Overall: n (%)	79(100.0)	39(49.4)	40(50.6)	
Mean patient age, y (range)	60.6(44.9-72.7)	60.4(47.6-68.2)	60.6(44.9-72.7)	0.772^a^
PSA Level: n (%)				0.219^b^
4	6(7.6)	4(10.3)	2(5.0)	
4-10	44(55.7)	23(60.0)	21(52.5)	
10	29(36.7)	12(30.7)	17(42.5)	
Gleason Score: n (%)				0.874^b^
<7	17(21.5)	10(25.6)	7(17.5)	
7	44(55.7)	19(48.8)	25(62.5)	
>7	18(22.8)	10(25.6)	8(20.0)	
N stage: n (%)				0.116^c^
N0	76(96.2)	36(92.3)	40(100.0)	
N1+	3(3.8)	3(7.7)	0	
T stage: n (%)				0.442^b^
≤T2a	50(63.3)	26(66.7)	24(60.0)	
T2b	20(25.3)	10(25.6)	10(25.0)	
≥T2c	9(11.4)	3(7.7)	6(15.0)	
Capsular Invasion: n (%)				0.009^c^
None	17(21.5)	10(25.6)	7(17.5)	
Focal	6(7.6)	1(2.6)	5(12.5)	
Invasive	18(22.8)	11(28.2)	7(17.5)	
Established	38(48.1)	17(43.6)	21(52.5)	
Extracapsular Extension:n(%)				0.056^c^
Negative	36(45.6)	22(56.4)	14(35.0)	
Positive	43(54.4)	17(43.6)	26(65.0)	
Seminal Vesicle Invasion				0.966^c^
No	69(87.3)	34(87.2)	35(87.5)	
Yes	10(12.7)	5(12.8)	5(12.5)	
Recurrence Status: n (%)				0.054^c^
NO	42(53.2)	25(64.1)	17(42.5)	
YES	37(46.8)	14(35.9)	23(57.5)	

^a^ T-test, ^b^ Wilcoxon rank sum test, ^c^ chi-square test.

**Table 3:** Univariate and multivariate Cox regression analysis of BCR of glinsky prostate dataset

	Univariate COX regression		Multivariate Cox regression	
	HR (95% CI)	*p* Value	HR (95% CI)	*p* Value
Age^*^, years		0.048		0.266
61.2	1.00 (referent)		1.00 (referent)	
≥61.2	1.956(1.006-3.805)		1.496(0.735-3.045)	
PSA Level		0.026		0.206
10	1.00 (referent)		1.00 (referent)	
≥10	2.107(1.103-4.024)		1.631(0.764-3.479)	
Gleason Score		0.000		0.010
≤7	1.00 (referent)		1.00 (referent)	
7	3.388(1.716-6.686)		2.883(1.292-6.433)	
N stage		0.033		0.062
N0	1.00 (referent)		1.00 (referent)	
N1+	3.704(1.113-12.323)		5.124(0.923-28.463)	
T stage		0.416		0.338
≤T2b	1.00 (referent)		1.00 (referent)	
≥T2c	1.483(0.575-3.827)		1.681(0.581-4.871)	
Seminal Vesicle Invasion		0.001		0.509
No	1.00 (referent)		1.00 (referent)	
Yes	3.825(1.742-8.400)		1.421(0.500-4.038)	
PDLIM5 expression		0.041		0.007
Low	1.00 (referent)		1.00 (referent)	
High	2.002(1.028-3.898)		2.792(1.316-5.927)	

^*^ Divided at median.

### Lentivirus-mediated knockdown and ectopic expression efficiency of PDLIM5 gene in PCa cell lines

**Figure 3:**
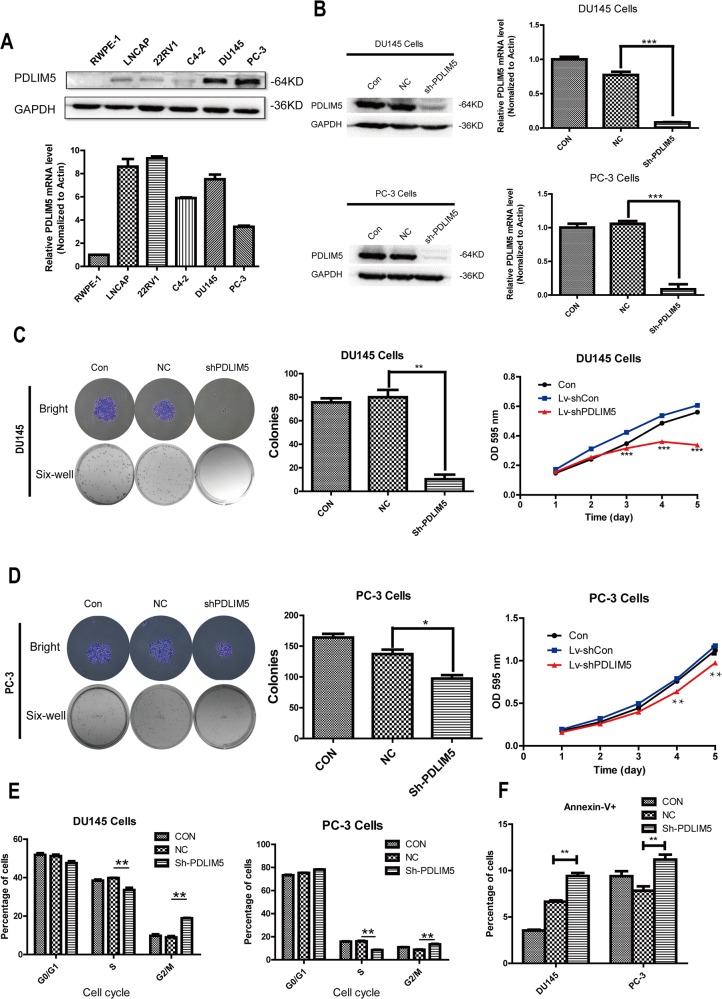
Silencing PDLIM5 inhibited colony formation and proliferation, and induced G2/M phase arrest and apoptosis in DU145 and PC-3 cells **(A)** The expression level of PDLIM5 in PCa cell lines by Western blotting and qPCR. **(B)** The silencing efficiency of PDLIM5 was assessed by qPCR and Wstern blot in DU145 and PC-3 cells. **(C, D)** The size and number of colonies for DU145 cells were significantly smaller in shPDLIM5 group as compared with shCON group, and the same result was in PC-3 cells. MTT assay: DU145 cell proliferation was significantly inhibited in shPDLIM5 group after 3-day culture, and the similarly result was obtained after 4-day culture in PC-3 cells. **(E)** The percentage of cell cycle distribution and apoptosis was analyzed using flow cytometry. Statistical analysis showed that both DU145 and PC-3 cells were arrested at G2/M phase in shPDLIM5 group as compared with shCON group. **(F)** The number of apoptotic cells stained by Annexin-V in shPDLIM5 group was significantly greater than that in the control group. Representative of three independent experiments. (^*^P< 0.05, ^**^P< 0.01; ^***^P< 0.001).

To investigate the function of PDLIM5, we first compared PDLIM5 expression between normal prostate cells (RWPE-1) and PCa cell lines LNCaP, 22RV1, C4-2, DU145 and PC-3 by qPCR and Western blotting. As shown in Figure 3A, PDLIM5 gene was up-regulated in PCa cell lines compared with that in RWPE-1 cells. Western blotting analysis showed that PDLIM5 protein was especially overexpressed in DU145 and PC-3 androgen independent cell lines. Therefore, DU145 and PC-3 were chosen to analyze lentivirus-mediated gene knockdown. To ensure the consistency of the experiment, PC-3 and DU145 were still used as ectopic expression cell lines. As represented by the percentage of GFP-expression, DU145 and PC-3 cells with a transduction efficiency of about 90% cells were subjected to functional analysis (Supplementary Figure 1A). Western blot and qRT-PCR indicated an efficient knockdown (Figure 3B) and ectopic express (Figure 5C) PDLIM5 in the cell lines.

### Silencing of PDLIM5 inhibits proliferation and colony formation by inducing G2/M arrest and apoptosis in DU145 and PC-3 cells

Compared with the control group, plate colony formation assay showed that the size and number of colonies were significantly reduced in PDLIM5 knockdown DU145 and PC-3 cells. DU145 and PC-3 cell proliferation was evaluated by MTT, showing that it was decreased after sh-PDLIM5 transduction (^**^P<0.001,^***^P<0.0001) (Figure 3C and 3D). In addition, FACS was performed to evaluate cell cycle distribution and assess the underlying mechanism of proliferation inhibition (Supplementary Figure 2A and 2B). It was found that PDLIM5 silencing induced G2/M phase arrest and apoptosis (Figure 3E and 3F), implying that the growth inhibition might be associated with increased cell cycle arrest and apoptosis. These results strongly support that PDLIM5 played an important role in PCa cell proliferation. Due to its involvement in cytoskeleton formation, PDLIM5 may affect the function of microfilaments and microtubules, leading to cell disruption, resulting in cell cycle arrest at G2 / M.

### Inhibition of PDLIM5 reduces invasion and migration of PCa cells

**Figure 4:**
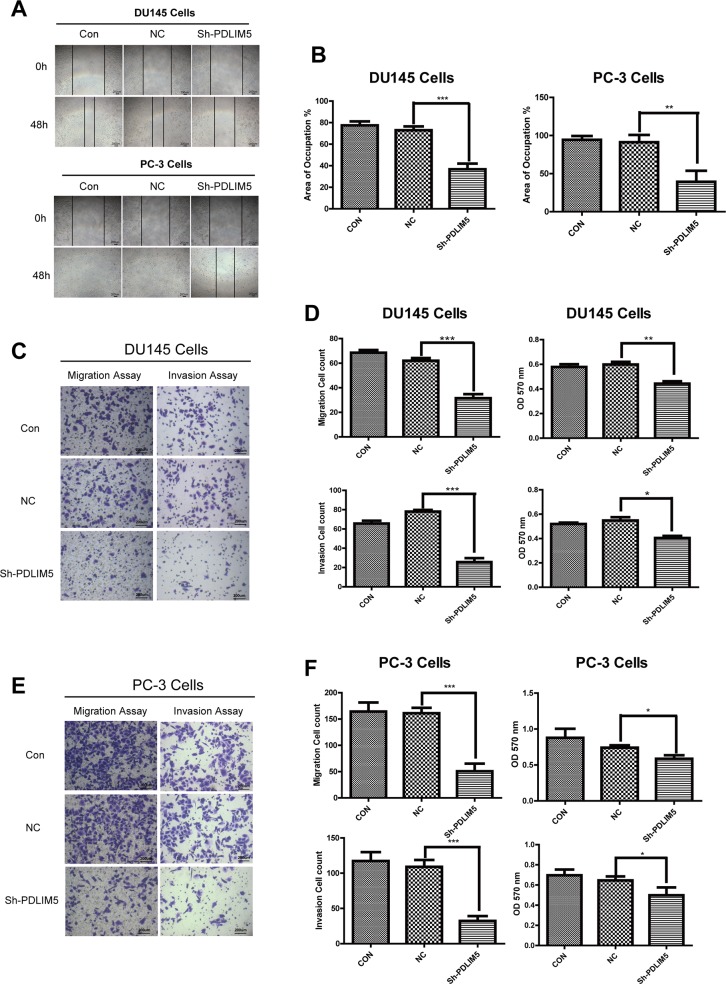
PDLIM5 knockdown inhibited PCa cell migration and invasion **(A)** The wound healing assays showed that the migration capability of DU145 and PC-3 in PDLIM5-silenced group was dramatically decreased compared with the control group. **(B)** The wound healing areas of DU145 and PC-3 were significantly smaller after PDLIM5 silencing than those in the control group (magnification X40, scale bar 200 um). **(C, E)** Transwell migration and invasion assay: Compared with the control group, the number of cells migrating through the 8um diameter pores and invading the matrigel membrane in shPDLIM5 group was decreased significantly. **(D, F)** DU145 and PC-3 cell counts in shPDLIM5 group were significantly lower than those in shCON group. The absorbance of shPDLIM5 group at 570 nm was significantly lower than that in the control group. Representative of three independent experiments. (^*^P< 0.05, ^**^P< 0.01; ^***^P< 0.001).

Cytoskeletal proteins may affect cell motility and migration. To explore the effect of PDLIM5 on metastasis and invasion of tumor cells, we first designed scratch-wound assays to evaluate the migration and invasion ability by calculating the migration of the two cell lines by the area covered by the migrated cells (Figure 4A and 4B). Knockdown of PDLIM5 in DU145 and PC-3 significantly inhibited the ability of migration (^**^P<0.001,^***^P<0.0001) compared with untreated cells. To further confirm the above results, a transwell migration and invasion assay was performed after 24-h starvation in DU145 and PC-3 to clarify the effect of PDLIM5 gene inhibition on cell invasion and migration. The crystal violet staining images showed that the number of cells in shPDLIM5 group on the underside of the filters were lower than that in the other two control groups (Figure 4C and 4E): the mean cell count of DU145 and PC-3 in shPDLIM5 group was 31.4 and 50.4 by migration assay and 27.6 and 33.8 by invasion assay respectively, showing a statistically significant decrease (^*^P <0.05) by light microscopy of randomly selected different views. In addition, the number of viable cells at 570 nm was significantly in shPDLIM5 group as compared with that in the other two control groups (Figure 4D and 4F).

### PDLIM5 affects EMT-related pathways *in vitro*

**Figure 5:**
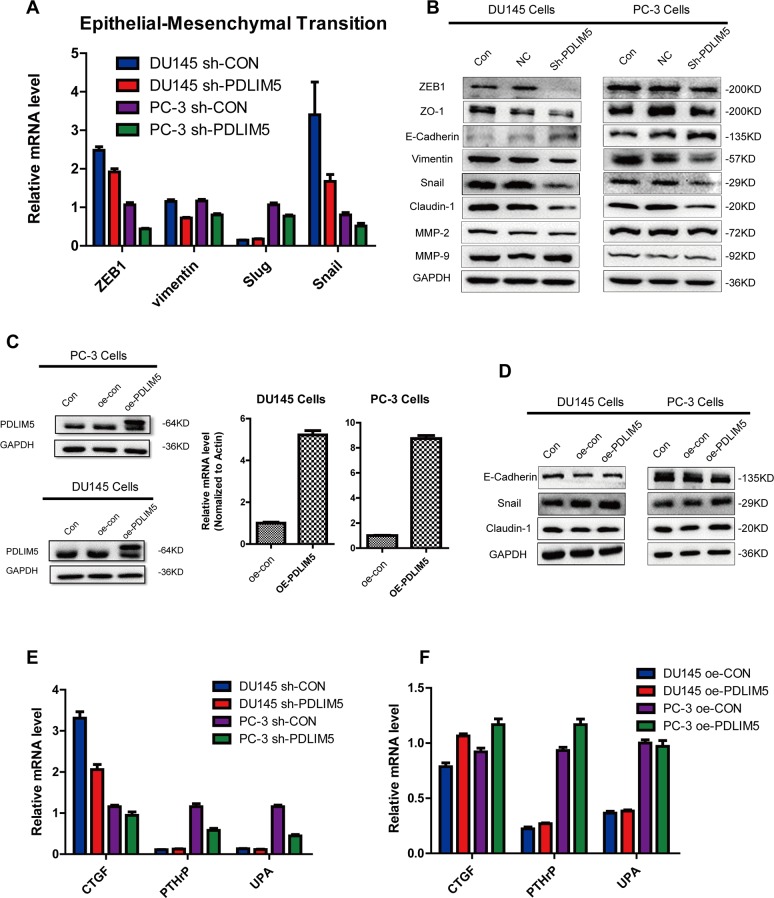
PDLIM5 regulates metastasis by EMT-related pathways **(A)** The expression level of EMT-related targets was decreased with PDLIM5 knockdown by qPCR analysis in DU145 and PC-3 cells. **(B)** Western blotting was used to analyze protein expression in EMT and of MMP proteins. **(C)** Ectopic expression of PDLIM5 in DU145 and PC-3 cells was detected by qPCR and Western blot. **(D)** The expression of snail/claudin-1 protein in EMT pathway changed after PDLIM5 overexpression. Each panel is representative of two independent experiments. **(E, F)** Changes in other invasion- and metastasis-related molecules after knocking down or overexpressing PDLIM5 by qPCR analysis.

Further study on the mechanism of inhibition of metastasis and invasion showed that PDLIM5 could affect the expression of EMT related pathway genes. After knocking down PDLIM5, the mRNA and protein expression levels of ZEB1, vimentin, snail and other EMT pathway molecules were downregulated, while E-Cadherin was increased as shown by Western blotting (Figure 5A and 5B). Overexpression of PDLIM5 could to some extent reverse the above trend (Figure 5D), suggesting that the effect of PDLIM5 on cell invasion and metastasis was closely associated with EMT. On the other hand, we studied other related molecules such as the connective tissue growth factor (CTGF), parathyroid hormone-related peptide (PTHrP) and urokinase-type plasminogen activator (uPA), which are known to promote tumor metastasis. The result of qPCR showed that after silencing PDLIM5, the expression of these pro-tumor metastasis factors in PC-3 cells was significantly down-regulated. Similarly, when PDLIM5was overexpressed, the expression levels of these molecules were up-regulated. The similar results were also observed in DU145 cells, further confirming that PDLIM5 played a role in promoting tumor metastasis.

### PDLIM5 can directly interact with AMPK protein to maintain its activation and reduce AMPK degradation

**Figure 6:**
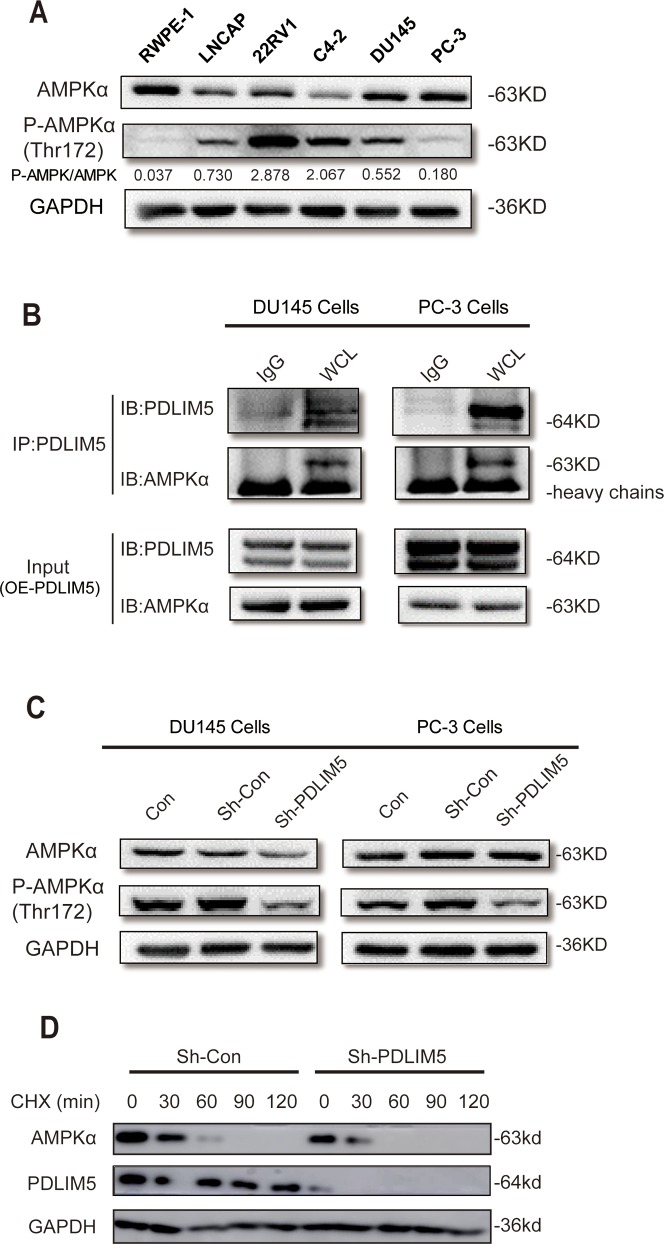
PDLIM5 maintains AMPK activation and decrease its degradation **(A)** The expression levels of AMPKα and P-AMPKα in PCa cell lines by Western blotting; the gray ratio of P-AMPKα/AMPKα blot is shown. **(B)** AMPKα protein directly bound to PDLIM5 in DU145 and PC-3 cells with ectopically expressed PDLIM5 **(C)** AMPKα phosphorylation level was significantly decreased after PDLIM5 knockdown. **(D)** Western blot assay for AMPKα expression level in DU145 cells with Sh-Con or PDLIM5 knockdown upon treatment of 50 μg/ml of cycloheximide (CHX) at various time points. Representative of two independent experiments.

**Figure 7:**
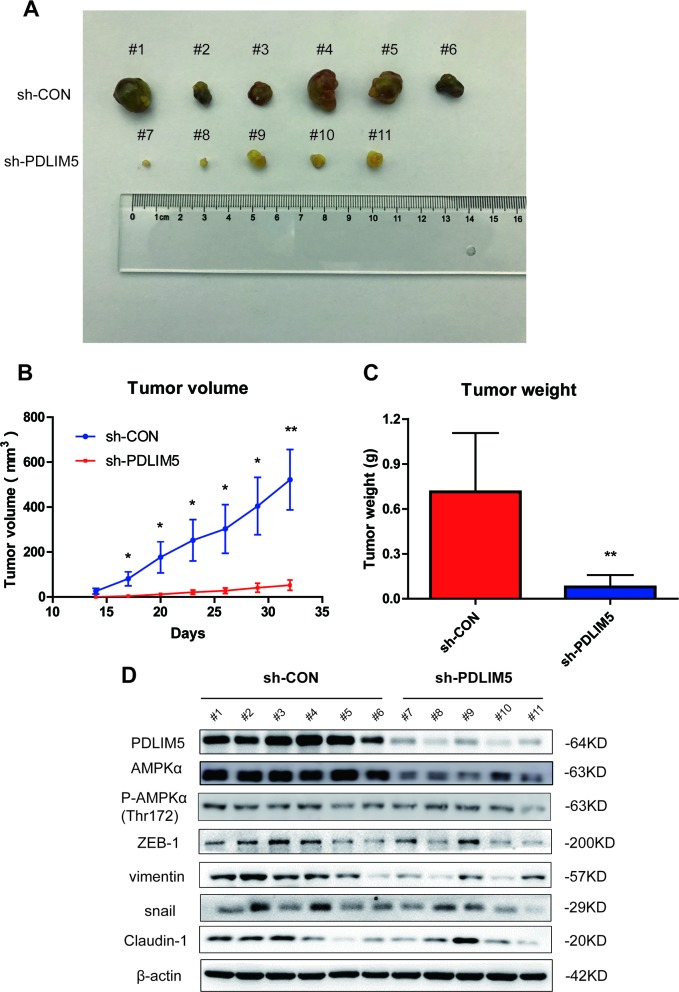
Knocking down PDLIM5 inhibits PCa cell growth by affecting AMPK and EMT signaling pathways *in vivo* **(A)** The subcutaneous xenograft tumor samples in nude mice were collected and imaged. **(B, C)** The tumor size and weight were significantly decreased in nude mice injected with shPDLIM5 DU145 cells (n=5) compared with mice with shCon DU145 cell injected group (n=6). **(D)** Western blotting was used to confirm PDLIM5 knockdown efficiency and analyze AMPKα expression and activation levels *in vivo*, and the protein expression in EMT pathway changed after PDLIM5 knockdown.

AMPK phosphorylate levels in PCa cell lines were measured and compared with those in normal prostate cells by Western blot. It was found that AMPK activation in PCa cells was higher than that in normal prostate epithelial cells (RWPE-1), suggesting that AMPK activation played an important role in the maintenance of PCa cell growth (Figure 6A). The CO-IP assay indicated that PDLIM5 could bind directly to AMPK (Figure 7B). To determine whether their combination had a physiological function, Western blotting was conducted to confirm the phosphorylate level and degradation of AMPK in shCON or shPDLIM5 group. Finally, PDLIM5 knockdown decreased the phosphorylation level in DU145 and PC-3 cells (Figure 6C), and induced more rapid degradation of AMPK protein upon cycloheximide treatment as compared with the control in DU145 (Figure 6D).

### Knockdown of PDLIM5 inhibits PCa cell growth and EMT *in vivo*

To further validate the oncogenic function of PDLIM5 *in vivo*, the tumor volume and weight were measured in both shCON and shPDLIM5 DU145 groups after subcutaneous tumor formation. The tumor size and weight in shPDLIM5-infected mice were significantly reduced as compared with those in Con group (Figure 7A-7C). We also used tumor tissues to confirm the effect of PDLIM5 knockdown *in vivo* by Western bolting. After PDLIM5 knockdown, AMPK activation was inhibited, degradation was increased, and ZEB1, vimentin, snail and Claudin-1 were downregulated as shown by Western blot (Figure 7D). These results further support the role of PDLIM5 as an oncogene in reducing tumor invasion *in vivo* by regulating AMPK activation and reversing EMT.

## DISCUSSION

Most currently available studies show that PDLIM5 and its family members can interact with a variety of proteins through PDZ and LIM domains, but the molecular species and the function of these interactions remain poorly understood [22–25]. Different PDLIM protein family members have regulatory effects in both cell-cell and cell-matrix interactions and migration. However, when PDLIM proteins become dysfunctional, they cannot support the maintenance of organ function, nor can they promote cancer cell Invasion and metastasis [26–28]. Immunohistochemical analysis and ONCOMINE data mining of the present study showed that the expression of PDLIM protein in PCa tissues was abnormally higher than that in normal prostate tissues, and the abnormally increased expression of PDLIM5 was associated with Gleason score, serum PSA level, metastasis and invasion and therefore may prove to be an independent risk factor for BCR in increasing the overall mortality. PDLIM5 was also upregulated in PCa cell lines as compared with normal prostate cells RWPE-1, particularly in non-hormone dependent cell lines PC-3 and DU145, indicating that PDLIM5 plays a key role in the progression of PCa.

Subsequently, through lentiviral vector encoding siRNA against PDLIM5 infected DU145 cells and PC-3 cells, we found that PDLIM5 knockdown inhibited PCa cell proliferation, blocked G2/M and induced PCa cell apoptosis. Long-term blockage of tumor cells in G2/M can inhibit cell growth, and at the same time a certain degree of DNA damage accumulation may also trigger tumor cell apoptosis [29], thus playing a role in the treatment of cancer. The filaments formed by microtubes are connected to the kinetochore of the chromosomes to ensure normal division of cells [30]. Meanwhile, PDLIM5 can act through stabilization of the cytoskeleton and its interaction with α-actin and integrin in regulating cell adhesion and maintaining cellular physiological functions [31]. Therefore, we hypothesize that PDLIM5 is necessary for functional microtubule formation, and knocking down PDLIM5 would damage the microtubule function and cause cell cycle and cell proliferation-related changes.

Tumor invasion and metastasis are not only the biological characteristics of malignant tumors but the important cause of cancer-related death. Studies have shown that the movement and invasion of cells are closely related to the dynamic changes of the cytoskeleton, especially the microfilament skeleton composed of actin [32]. PDLIM5 silencing caused abnormal function of the cytoskeleton, thus decreasing the ability of cell migration. Likewise, the absence of PDLIM5 in tumor cells inhibited cell invasion and metastasis.

Epithelial-mesenchymal transition is one of the key processes in malignant progression, which gives epithelial cancer cells the ability to break through the basement membrane and metastasize to distal sites [33]. EMT is characterized by the loss of E-cadherin in epithelial cell adhesion molecules that allow cells to increase exercise and aggressiveness by destroying cell-cell contacts [34]. Snail and slug, two key regulators of EMT, can bind to the CDH1 promoter region of the E-cadherin protein and downregulate its expression; loss of E-cadherin can increase the expression of other corresponding mesenchymal-related genes, including coding for vimentin and N-cadherin [35]. In this study, we investigated the expression of EMT-related molecules by Western blot and qPCR. PDLIM5 Knockdown downregulated the above-mentioned mesenchymal-related genes. Ectopic expression of PDLIM5 in DU145 and PC-3 cells could to some extent reverse the EMT phenotype, which further indicates that PDLIM5 is closely associated with PCa cell metastasis.

Similar to other solid tumors, micro-environmental changes also occur in PCa cells in the process of malignant proliferation and metastasis, resulting in hypoxia and metabolic stress [36]. AMPK can be activated under metabolic stress to maintain cell metabolism and proliferation in stress situations. It was reported [37, 38] that AMPK was in an active state in PCa, thus allowing PCa cells to maintain normal cellular function during malignant proliferation and metastasis. Excessive activation of AMPK was associated with PCa progression and promoted human PCa cell growth and survival [39]. Our above experiments demonstrated that PDLIM5 played diverse physiological functions in PCa cells. More importantly, PDLIM5 could bind to AMPK directly through the CO-IP method, and silencing PDLIM5 could decrease the phosphorylation of AMPK and promote the degradation of AMPK protein, suggesting that PDLIM5 may be an important ligand for AMPK to maintain structural stability. When PDLIM5 was knocked down, AMPK structural integrity may be impaired, which further impaired the corresponding phosphorylation and inhibited activation of the AMPK signaling pathway. AMPK is involved in a variety of cellular physiological functions, including participating in functional microtubule formation [40], autophagy [20] and EMT [41] management in cancer cells. These functions are closely related to cell cycle, apoptosis and metastasis in PCa cells. Therefore, we hypothesize that PDLIM5 plays an important role in PCa cells because of its interaction with AMPK, which stimulates the proliferation and metastasis of tumor cells by affecting the activation of AMPK. Meanwhile, PDLIM5 can also extend its function by remodeling the cytoskeleton.

In conclusion, the present study demonstrated that PDLIM5 was upregulated in PCa clinical specimens, suggesting that PDLIM5 may be an oncogene in PCa cells. To the best of our knowledge, this is the first study of using bioinformatics analysis to study the association of PDLIM5 with PCa progression, recurrence and metastasis. In addition, both *in vivo* and *in vitro* experiments demonstrated that the abnormally high expression of PDLIM5 increased the invasiveness of PCa cells. PDLIM5 could interact with AMPK and affect its activity, which is also an important reason why PDLIM5 showed diverse physiological activities. Identification of novel molecular pathways and targets with PDLIM5 may help better understand PCa progression and metastasis, and seek new treatment strategies for patients with mCRPC.

## MATERIALS AND METHODS

### Clinical specimens and immunohistochemistry

Altogether 52 clinical specimens were collected during radical resection for PCa at the department of urological surgery of Changzheng Hospital (Shanghai, China) between March 2012 and March 2014. The clinical characteristics of the 52 patients were analyzed retrospectively. The study was approved by the Ethics Committee of the Second Military Medical University (Shanghai, China). All patients were informed of the purpose of their specimens and provided informed consent. All specimens were fixed in formalin, embedded in paraffin, sliced to 4-μm thick sections, HE stained, and then evaluated histologically by a pathologist who was blind to the clinical data of the patients. Finally, 44 cases of cancer tissues were confirmed and paired paracarcinoma tissues were used as negative controls.

### Immunohistochemistry

The sections were dewaxed in xylene and different concentrations of ethanol, incubated with 1:50 rabbit anti-PDLIM5 (#:10530-1-AP, Proteintech) at 4°C overnight and then with general biotinylated goat anti-rabbit serum and streptavidin-peroxidase conjugate at room temperature for 15 min, and finally stained with diaminobenzidine.

### Cell culture

PCa cell lines DU145 and PC-3 were purchased from the Cell Bank of the Chinese Academy of Sciences (Shanghai, China). PC-3 cells were maintained in Ham's F-12 Nutrient Mixture (Gibico) with 10% fetal bovine serum (FBS), 100 units/mL penicillin, 100 μg/mL streptomycin. DU145 was cultured in the same way as PC-3 with addition of 1 mM nonessential amino acid. All cells were grown in a humidified incubator in a 5% CO2 atmosphere at 37°C.

### Lentivirus construction and transfection

Small interfering RNA (si-RNA) targeting PDLIM5 (NM_006457) and a scramble siRNA sequence were transformed into stem-loop-stem oligos (sh-RNAs). The targeting and scramble sh-RNA sequences were 5′-CTGTGTAAGAAACATGCTCAT-3′ and 5′-TTCTCCGAACGTGTCACGT-3′, respectively. The sh-RNAs were inserted into the pFH-L vector and the generated lentiviral based sh-RNA expressing vectors were confirmed by DNA sequencing. The sh-RNA expression vector and packaging vectors (pVSVG-I and pCMVMR8.92) were cotransfected into HEK293T cells with Lipofectamine 2000 according to the manufacturer's instructions. The supernatant was collected 48 h and 72 h later, centrifuged (4000 g, 4°C, 10 min) to remove cell debris, filtered through 0.45μm cellulose acetate filters, and then concentrated by ultracentrifugation (50000 g, 4°C,1h30min). DU145 and PC-3 cells (5 × 104 cells/well) were seeded in 6-well plates and infected with PDLIM5 sh-RNA (Lv-shPDLIM5) or control sh-RNA (Lv-shCon) expressing lentivirus at a multiplicity of infection (MOI) of 30 and 35 for 72 h. As the lentivirus carries a green fluorescence protein (GFP) reporter driven by the CMV promoter, the infection efficiency was measured by counting GFP-expressing cells under a fluorescence microscope (CKX41, Olympus). PDLIM5 gene knockdown efficiency was evaluated by Quantitative Real-Time PCR (qRT-PCR) and Western blotting.

### Quantitative real-time PCR

Total RNA was extract using 1ml Trizol reagent (Invitrogen) and each RNA purification process was performed according to manufacturer's instructions. The concentration of RNA extraction was determined using ultraviolet analysis and determined based on the ratio of A260/A280. 2 μg total RNA was reverse transcribed into cDNA using Oligo (dT) primer and the M-MLV Reverse Transcriptase (Promega). Quantitative real-time PCR (qPCR) reaction containing 10 μl 2 × SYBR Premix Ex Taq (Bio-Rad), 0.5 μl primer (Bio-Rad), 5 μl cDNA and 4.5 μl nuclease-free water was run on the ABI PRISM 7700 Sequence Detection System (Applied Biosystems, Foster City, CA). The primer sequences were used: for PDLIM5, 5′-TTAGTGGCACTGGGGAAATC-3′(forward) and 5′-GATCTTCCTTTGGCATCGAC-3′ (reverse); for ZEB1, 5′- CCTGCCAACAGACCAGACAG-3′ (forward) and 5′- CTTCAGGCCCCAGGATTTCTT -3′ (reverse) ;for Vimentin, 5′- TCACCTGTGAAGTGGATGCC -3′ (forward) and 5′- ACGAAGGTGACGAGCCATTT-3′ (reverse); for SLUG, 5′- TCGGACCCACACATTACCTTG -3′ (forward) and 5′- AAAAAGGCTTCTCCCCCGTG -3′ (reverse); for Snail, 5′- CCAGTGCCTCGACCACTATG -3′ (forward) and 5′- CTGCTGGAAGGTAAACTCTGG -3′ (reverse); for CTGF, 5′- CTCCTGCAGGCTAGAGAAGC -3′ (forward) and 5′- GATGCACTTTTTGCCCTTCTT -3′ (reverse) ; for PTHrP, 5′- GTCTCAGCCGCCGCCTCAA -3′ (forward) and 5′- GGAAGAATCGTCGCCGTAAA -3′ (reverse) ; for uPA, 5′- ATCTGCCTGCCCTCGATGTATAA -3′ (forward) and 5′- TTTCAGCTGCTCCGGATAGAGATAG -3′ (reverse); for β-actin, 5′-ATCGTGCGTGACATTAAGGAG-3′ (forward) and 5′-AGGAAGGAAGGCTGGAAGAG-3′ (reverse). The relative expression level was calculated using the 2-^ΔΔ^Ct method on the basis of β-actin for normalization. Each experiment sample was repeated in triplicate.

### Western blotting

Cells were homogenized in 0.2 ml ice cold lysis buffer (50 mM Tris-HCl, pH 7.4, 4% SDS, 20% Glycerol, 2% Mercaptoethanol). After sufficient lysis, the lysate was centrifuged at 12,000 rpm for 5 min at 4°C and the supernatant was collected. The protein concentration was determined using BCA assay. Proteins after lysis were separated in 10% SDS-PAGE gel and transferred onto PVDF membranes for immunoblotting analysis. Then, the membranes were soaked in 40-50ml TBST solution with 5% nonfat milk for 1 h at room temperature, and then incubated with primary antibodies: PDLIM5 (#:10530-1-AP, Proteintech); Epithelial-Mesenchymal Transition (EMT) Antibody Sampler Kit (#:9782, Cell Signaling)); AMPKα (#:5831, Cell Signaling); Phospho-AMPKα(Thr172) (#:2535, Cell Signaling);GAPDH (#:10494-1-AP, Proteintech) at 4°C overnight. After three washes with 40-50ml TBST solution, the transferred membranes were incubated with 1:5000 horseradish peroxidase-conjugated goat anti-rabbit IgG antibody (Santa Cruz Biotechnology, Santa Cruz, CA) for 1 h at room temperature. The protein signals were detected in ECL plus TM Western blotting system kit. GAPDH protein was used as internal reference standard.

### Co-immunoprecipitation assay

For identification of proteins associated with PDLIM5, 20 mg purified ectopic expression PDLIM5 was incubated at 4°C for 2 h with the cell lysate from DU145 and PC-3 cells in lysis buffer (30mM MOPS pH 7.5, 150mM NaCl, 0.5% Triton-X100, 1.5mM MgCl2, 1mM EGTA, 1mM DTT and protease inhibitor cocktail). Protein A agarose was prepared and the beads were washed twice with PBS. The supernatant was transferred to a new centrifuge tube, to which 100 μl Protein A agarose beads per 1 ml of total protein was added, shaken for 10 min at 4°C to remove nonspecific protein, and centrifuged at 12,000 rpm for 15 min at 4°C. The supernatant was transferred to a new centrifuge tube. The protein concentration was determined by BCA assay. 4ul AMPKα antibody was added to 500μg total protein and 4 μl rabbit anti-rabbit IgG for control group, and the antigen-antibody mixture was shaken gently overnight at 4°C. 100 μl Protein A agarose beads were used to capture the antigen-antibody complex for 1 h. The complex was centrifuged instantaneously at 12,000 rpm for 5s. The agarose bead-antigen antibody complex was collected, washed three times with pre-cooling PBS, and detected for PDLIM5 and AMPKα antibodies by Western blotting.

### Cell proliferation assay

After 5-day infection, cell viability was evaluated by 3-(4,5)-dimethylthiahiazo (-z-y1)-3,5-di- phenytetrazoliumromide (MTT, Sigma-Aldrich) assay. The treated cells were trypsinized, suspended, counted, and plated in a 96-well plate (five wells for each group) at a density of 2 × 103 cells/well for 1, 2, 3, 4 and 5 days. The cells in the 96-well plate were incubated for 4 h. 20μl MTT solution (5mg/ml) was added into each well for 3 h, and then 100μl acidified isopropanol was added into each well to terminate MTT reaction. The absorbance of each well was determined at 570 nm using the spectrophotometer (ELx808 Bio-Tek Instruments, USA).

### Cell colony formation assay

After 5-day infection, treated cells were trypsinized, suspended, counted and plated in a 96-well plate (three wells for each group) at a density of 200 cells/well. The cells in the 96-well plate were incubated for 7-14 days, with the culture medium replaced every 3 days. Afterwards, the cells in the 96-well plate were fixed in 4% paraformaldehyde for 30 min and stained by GIEMSA dye (Sigma) for 20 min. The number of cell colonies was counted under a microscopy and each plate was photographed with a digital camera.

### Cell cycle and apoptosis analysis

After 5-day infection, cells were trypsinized, centrifuged at 1200rpm for 3 min. For cell cycle analysis the cells were washed with ice-cold PBS, and fixed with 950μL ice-cold 75% ethanol for 1 h. The fixed cells were washed with ice-cold PBS, centrifuged at 1500rpm for 5 min and incubated with 500μL freshly prepared PI staining solution containing PI dye and RNase A (Sigma) at 37°C in dark for 1 h. For cell apoptosis analysis cells were resuspended with Binding Buffer. Annexin-V/ 7-ADD (BD Biosciences, USA) double staining was performed at 4°C in dark for 30min. Each experiment was repeated in triplicate. Cell cycle distribution and apoptosis count was analyzed by flow cytometry using a dedicated software (FACS Calibur, BD, San Jose, CA).

### Scratch-wound migration assays

Scratch-wound migration assays were preformed as well. Both DU145 and PC-3 were seeded in 96-well plates (five wells for each group) after 72-h infection with the virus. Each well had PC-3 and DU145 about 3×10^4^ and 2×10^4^ respectively. Cells were maintained in the F12 Medium with 10% serum for 24 h. And then the medium was changed to 2% serum F12 after using 200ul pipette tip scratched per well. Each well was observed at a 24-h interval, and photographed after 72 h. The images were taken with an inverted microscope at 40X magnification (Nikon Co. Japan). Every well was calculated by the ImageProPlus 6.0.

### Transwell invasion assay

After 24-h infection, cells were trypsinized, centrifuged at 1500 rpm for 5 min, washed with PBS, and resuspended with 0.1% BSA. Cells were seeded into the upper compartment with the matrigel membrane and incubated in a cell incubator for 24 h. Cells in the upper compartment were removed using a moist cotton swab and washed with PBS. Cells in the lower compartment of the matrigel membrane were fixed with 10% methanol solution for 30 seconds, stained with 0.15 crystal violet for 20 min, and washed with ddH2O. Each experiment was repeated in triplicate. Cells were counted and observed under a light microscope.

### Animal experiments

Twelve six-week-old nude mice were randomly divided into two groups and then injected subcutaneously on the right flank with 5×10^6^ DU145 cells pre-infected with shCon and shPDLIM5 lentiviruses. Two weeks later, the tumor size was measured every three days and calculated using the formula: (length × width^2^)/2. At the end of the experiment, the mice were euthanized and the tumors were removed from each nude mouse, imaged and weighed. Tumor tissues were preserved at −80°C for WB detection.

### Oncomine and PROGgene database for bioinformatic analysis

A set of microarray data were analyzed with the Oncomine (http://www.oncomine.org) and PROGgene (watson.compbio.iupui.edu/chirayu/proggene/database) databases. The data were filtered by “PDLIM5”, “PCa and normal tissues” and the data type was defined as mRNA. Then 12 datasets were acquired, and a meta-analysis was carried out to compare the level of the PDLIM5 between the PCa and the normal gland tissue. Besides, we compared the expression of PDLIM5 in tumors with different Gleason scores, primary lesions and metastatic lesions. The information about biochemical recurrence and overall survival (OS) in Glinsky Prostate, GSE40272 and TCGA database was obtained for survival analysis. All data were reported as Log2 Median-Centered intensity in the ONCOMINE or PROGgene microarray datasets and processed by GraphPad Prism 5.

### Statistical analysis

The data were shown as mean ± standard deviation (SD) of at least three experiments. Pearson Chi-square Test and Mann-Whitney U were used to analyze PDLIM5 expression level in PCa and adjacent tissues. The Kaplan-Meier analysis was used to evaluate the relation between the prognosis and the PDLIM5 expression in microarray datasets. Statistical comparison of the shCon and shPDLIM5 was performed using Student t-test. The SPSS version 20.0 (IBM Corporation, USA) was used to calculate the statistics, and P-value<0.05 was considered statistically significant.

## SUPPLEMENTARY MATERIALS FIGURES AND TABLES


