# Supplementary material for: High expression of PDLIM5 facilitates cell tumorigenesis and migration by maintaining AMPK activation in prostate cancer
Source: Oncotarget. 2017 Sep 18;8(58):98117–34. doi: 10.18632/oncotarget.20981 (PMC5716718; doi:10.18632/oncotarget.20981)
Supplement: Supplementary file 1 [file oncotarget-08-98117-s001.pdf]

High expression of PDLIM5 facilitates cell tumorigenesis and migration by maintaining AMPK activation in prostate cancer

SUPPLEMENTARY MATERIALS

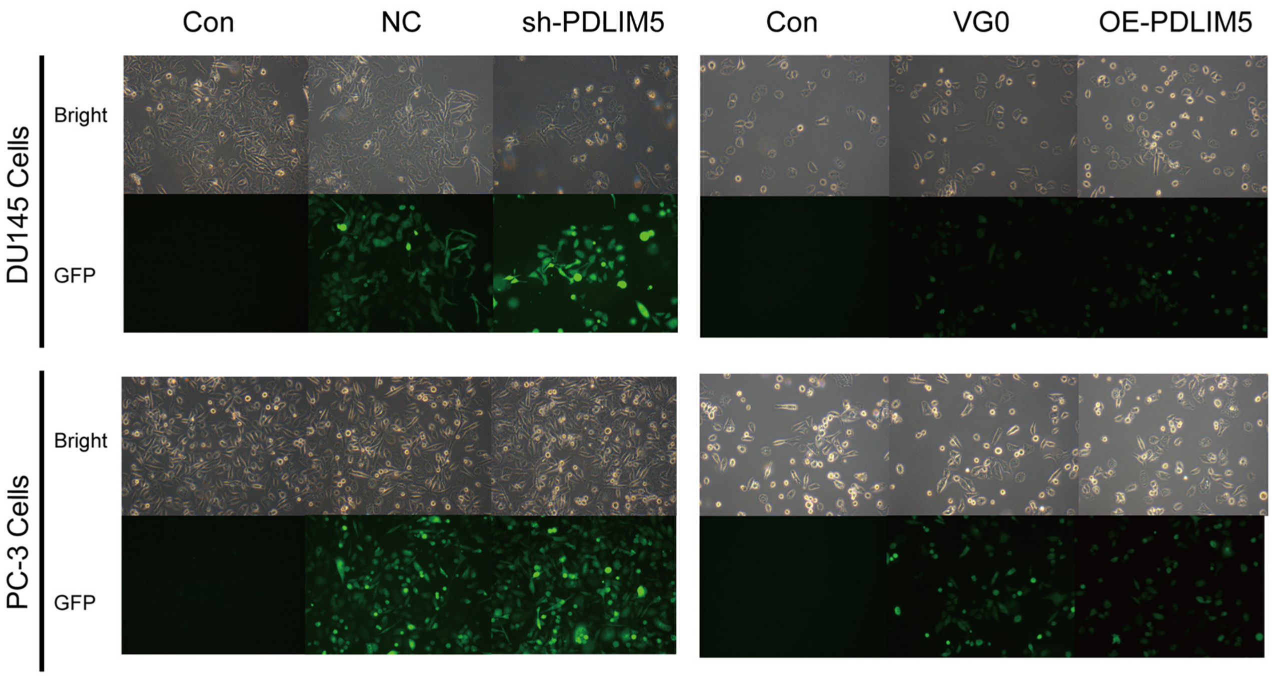

Supplementary Figure 1: Lentivirus-mediated knockdown and overexpress of PDLIM5 in DU145 and PC-3. The lentivirus was successfully transfected and the result was measured in in bright and GFP (magnification ×100).

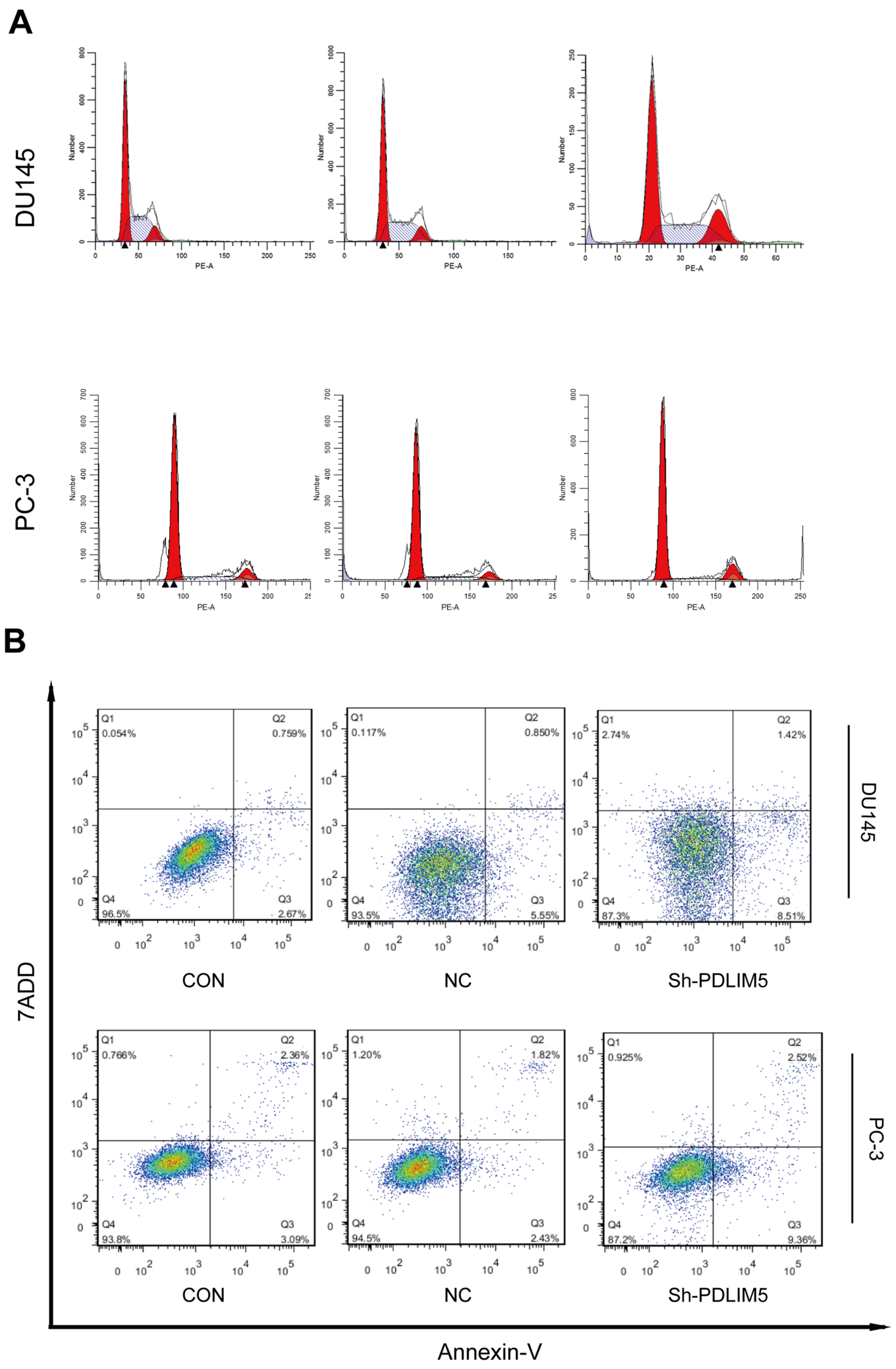

Supplementary Figure 2: Cell cycle distribution **(A)** and apoptosis **(B)** of DU145 and PC-3 cells were analyzed using flow cytometry and annexin V/7ADD staining in Con, shCon and shPDLIM5 groups.

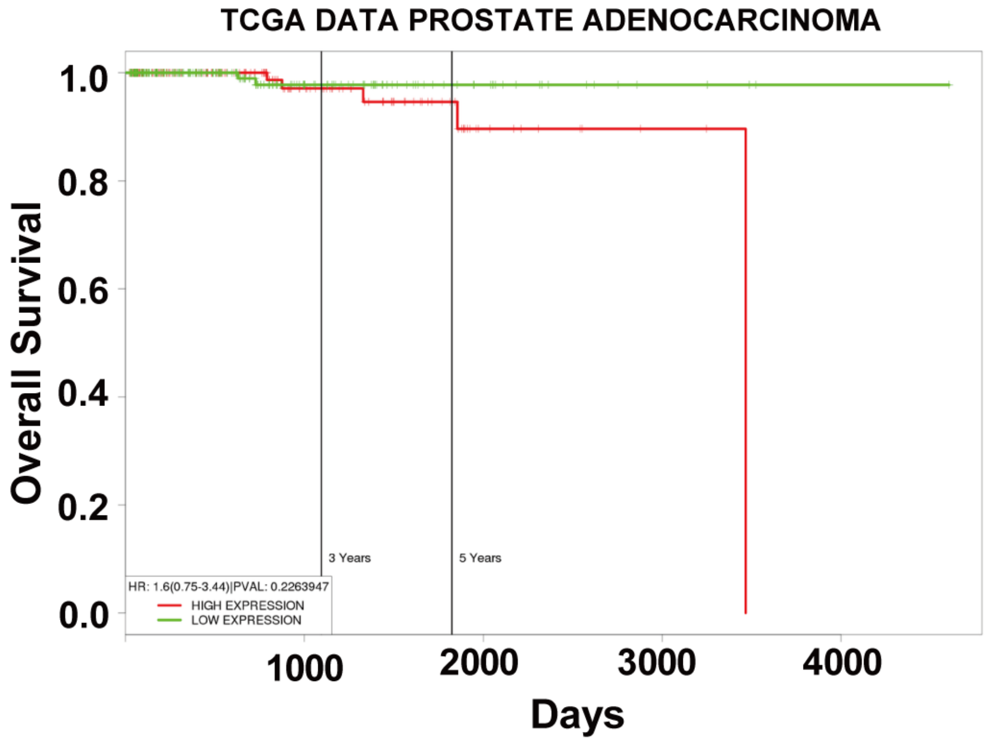

Supplementary Figure 3: High expression of PDLIM5 reducing OS was conclude by TCGA database (PROGGENE). Hazard ratio (HR) and P-value are shown in the image.
